# 3-[(1-Bromo­naphthalen-2-yl)meth­oxy]-5,5-di­methyl­cyclo­hex-2-enone

**DOI:** 10.1107/S160053681301026X

**Published:** 2013-04-20

**Authors:** Liang-Liang Fang, Nan Liu, Xin-Ying Zhang

**Affiliations:** a School of Chemistry and Chemical Engineering, Henan Normal University, Henan 453007, People’s Republic of China

## Abstract

In the title compound, C_19_H_19_BrO_2_, the cyclo­hexenone ring adopts an envelope conformation with the C atom bearing the methyl substituents as the flap. In the crystal, weak π–π stacking is observed between parallel aromatic rings of adjacent mol­ecules, the centroid–centroid distance being 3.694 (6) Å. The entire bromonaphthylmethyl unit is disordered over two orientations, with a site-occupancy ratio of 0.5214 (19):0.4786 (19).

## Related literature

For the biological activity and applications of cyclo­hex-2-enone derivatives, see: Aghil *et al.* (1992 ▶); Correcia *et al.* (2001 ▶); Ghorab *et al.* (2011 ▶).
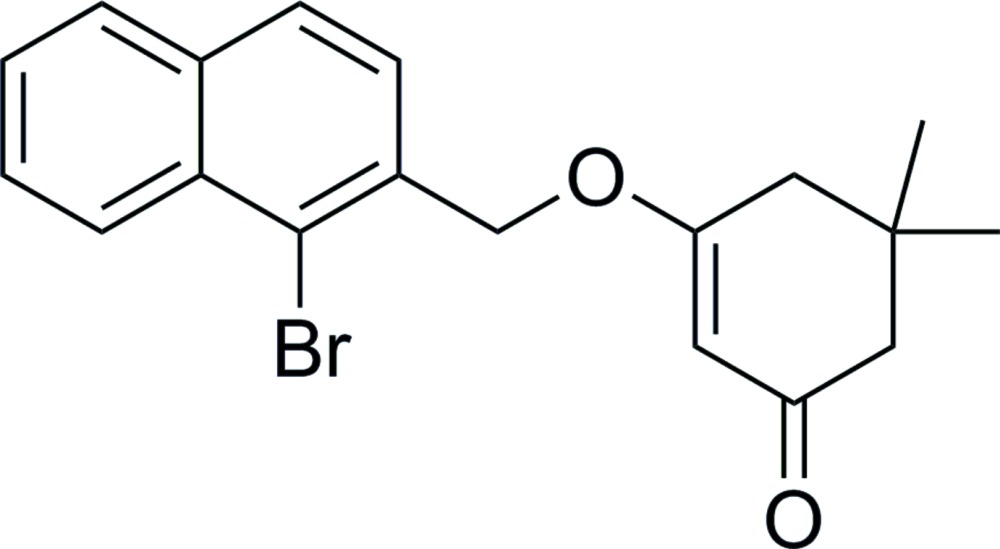


## Experimental

### 

#### Crystal data

C_19_H_19_BrO_2_
*M* *_r_* = 359.25
Monoclinic, 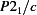

*a* = 13.986 (3) Å
*b* = 9.9970 (18) Å
*c* = 11.859 (2) Å
β = 91.169 (2)°
*V* = 1657.8 (5) Å^3^
*Z* = 4
Mo *K*α radiation
μ = 2.48 mm^−1^
*T* = 296 K
0.32 × 0.29 × 0.27 mm

#### Data collection

Bruker SMART 1000 CCD area-detector diffractometer
Absorption correction: multi-scan (*SADABS*; Bruker, 2001 ▶) *T*_min_ = 0.504, *T*_max_ = 0.554
11934 measured reflections
3075 independent reflections
1931 reflections with *I* > 2σ(*I*)
*R*_int_ = 0.074

#### Refinement

*R*[*F* ^2^ > 2σ(*F* ^2^)] = 0.061
*wR*(*F* ^2^) = 0.199
*S* = 1.07
3075 reflections
222 parameters
72 restraints
H-atom parameters constrained
Δρ_max_ = 0.26 e Å^−3^
Δρ_min_ = −0.20 e Å^−3^

### 

Data collection: *SMART* (Bruker, 2007 ▶); cell refinement: *SAINT* (Bruker, 2007 ▶); data reduction: *SAINT*; program(s) used to solve structure: *SHELXS97* (Sheldrick, 2008 ▶); program(s) used to refine structure: *SHELXL97* (Sheldrick, 2008 ▶); molecular graphics: *SHELXTL* (Sheldrick, 2008 ▶); software used to prepare material for publication: *SHELXTL*.

## Supplementary Material

Crystal structure: contains datablock(s) I, global. DOI: 10.1107/S160053681301026X/xu5694sup1.cif

Structure factors: contains datablock(s) I. DOI: 10.1107/S160053681301026X/xu5694Isup2.hkl

Supplementary material file. DOI: 10.1107/S160053681301026X/xu5694Isup3.cdx

Supplementary material file. DOI: 10.1107/S160053681301026X/xu5694Isup4.cml

Additional supplementary materials:  crystallographic information; 3D view; checkCIF report

## Figures and Tables

**Fig. 1.**
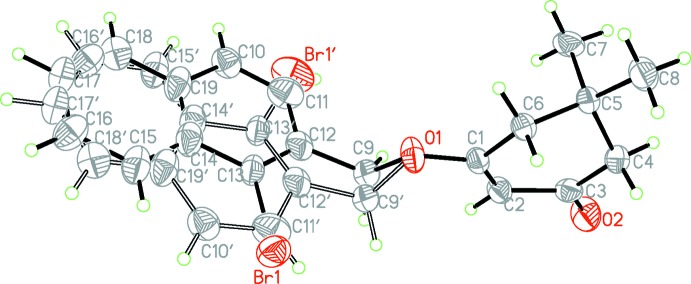
Molecular structure of the title compound with the disorder atoms, with displacement ellipsoids drawn at the 30% probability level.

**Fig. 2.**
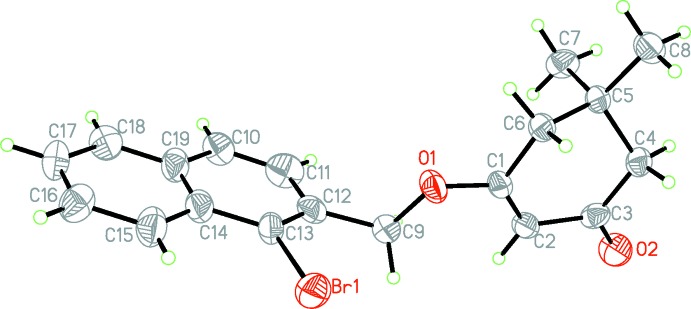
Molecular structure of the title compound without the disorder atoms, with displacement ellipsoids drawn at the 30% probability level.

**Fig. 3.**
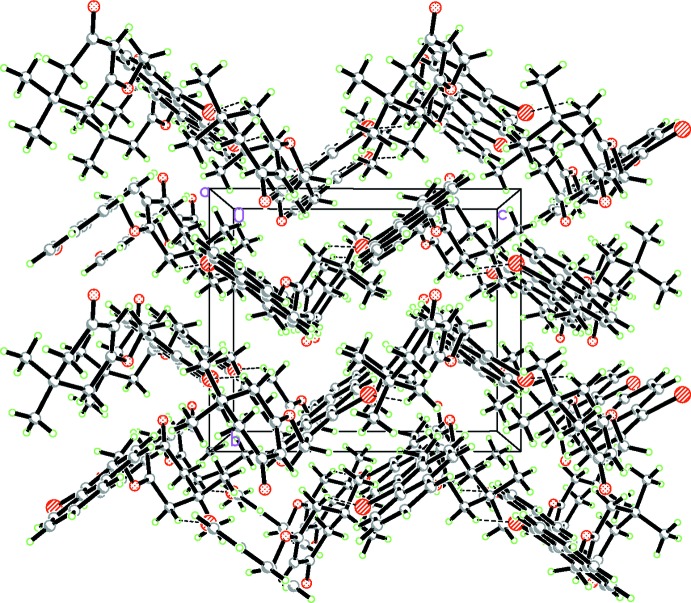
Crystal structure of the title compound with view along the a axis (disorder atoms have been omitted for clarity).

## supplementary crystallographic information

### Comment

Cyclohex-2-enone derivatives display a wide range of biological activities (Aghil *et al.*, 1992; Correcia *et al.*, 2001). Moreover, they have been frequently used as precursors in the synthesis of heterocyclic compounds (Ghorab, *et al.*, 2011). The title compound is the derivative of cyclohex-2-enones, and we report its crystal structure here.

In the title compound, C_19_H_19_BrO_2_, all the bond lengths and bond angles are within normal ranges. The cyclohexenone ring adopts an envelope conformation with the C atom bearing two methyl groups as the flap atom. All the atoms in the *o*-bromonaphthylmethyl group are disordered over two positions with site occupancy factors of 0.521 (2) and 0.479 (2).

In the crystal structure, weak π-π stacking is observed between parallel aromatic rings of adjacent molecules, the centrods distance being 3.694 (6) Å.

### Experimental

To a solution of 1-bromo-2-(bromomethyl)naphthalene (0.15 g, 0.5 mmol) and 5,5-dimethylcyclohexane-1,3-dione (0.14 g, 1.0 mmol) in DMF (3 ml) were added K_2_CO_3_ (0.21 g, 1.5 mmol) and CuI (0.01 g, 0.05 mmol). The mixture was stirred at 373 K until a complete conversion. It was cooled to room temperature and added with water, then extracted with ethyl ether (5 ml × 3). The combined organic phases were concentrated under vacuum. The crude product was purified by column chromatography eluting with ethyl acetate/hexane (10–20%) to give the title compound with the yield of 32% (0.057 g, 0.16 mmol). Single crystals, suitable for X-ray diffraction analysis, were obtained by slow evaporation of the solvents from a chloroform-ethyl acetate (1:1 *v*/*v*) solution of the title compound.

### Refinement

H atoms were positioned geometrically and refined using riding model with C—H = 0.93–0.97 Å, *U*_iso_(H) = 1.2*U*_eq_(C). The The bromonaphthalene moiety is disordered over two orientations, the site occupancies were refined to 0.5214 (19):0.4786 (19), the ADP of corresponding atoms in the disordered components were restrained as the same.

### Crystal data

**Table:**

C_19_H_19_BrO_2_	*F*(000) = 736
*M**_r_* = 359.25	*D*_x_ = 1.439 Mg m^−^^3^
Monoclinic, *P*2_1_/*c*	Mo *K*α radiation, λ = 0.71073 Å
Hall symbol: -P 2ybc	Cell parameters from 2538 reflections
*a* = 13.986 (3) Å	θ = 2.5–20.6°
*b* = 9.9970 (18) Å	µ = 2.48 mm^−^^1^
*c* = 11.859 (2) Å	*T* = 296 K
β = 91.169 (2)°	Block, colourless
*V* = 1657.8 (5) Å^3^	0.32 × 0.29 × 0.27 mm
*Z* = 4	

### Data collection

**Table:**

Bruker SMART 1000 CCD area-detector diffractometer	3075 independent reflections
Radiation source: fine-focus sealed tube	1931 reflections with *I* > 2σ(*I*)
Graphite monochromator	*R*_int_ = 0.074
phi and ω scans	θ_max_ = 25.5°, θ_min_ = 2.5°
Absorption correction: multi-scan (*SADABS*; Bruker, 2001)	*h* = −16→16
*T*_min_ = 0.504, *T*_max_ = 0.554	*k* = −12→12
11934 measured reflections	*l* = −14→14

### Refinement

**Table:**

Refinement on *F*^2^	Primary atom site location: structure-invariant direct methods
Least-squares matrix: full	Secondary atom site location: difference Fourier map
*R*[*F*^2^ > 2σ(*F*^2^)] = 0.061	Hydrogen site location: inferred from neighbouring sites
*wR*(*F*^2^) = 0.199	H-atom parameters constrained
*S* = 1.07	*w* = 1/[σ^2^(*F*_o_^2^) + (0.0635*P*)^2^ + 1.8223*P*] where *P* = (*F*_o_^2^ + 2*F*_c_^2^)/3
3075 reflections	(Δ/σ)_max_ < 0.001
222 parameters	Δρ_max_ = 0.26 e Å^−^^3^
72 restraints	Δρ_min_ = −0.20 e Å^−^^3^

### Special details

**Table:**

Geometry. All e.s.d.'s (except the e.s.d. in the dihedral angle between two l.s. planes) are estimated using the full covariance matrix. The cell e.s.d.'s are taken into account individually in the estimation of e.s.d.'s in distances, angles and torsion angles; correlations between e.s.d.'s in cell parameters are only used when they are defined by crystal symmetry. An approximate (isotropic) treatment of cell e.s.d.'s is used for estimating e.s.d.'s involving l.s. planes.
Refinement. Refinement of *F*^2^ against ALL reflections. The weighted *R*-factor *wR* and goodness of fit *S* are based on *F*^2^, conventional *R*-factors *R* are based on *F*, with *F* set to zero for negative *F*^2^. The threshold expression of *F*^2^ > σ(*F*^2^) is used only for calculating *R*-factors(gt) *etc*. and is not relevant to the choice of reflections for refinement. *R*-factors based on *F*^2^ are statistically about twice as large as those based on *F*, and *R*- factors based on ALL data will be even larger.

### Fractional atomic coordinates and isotropic or equivalent isotropic displacement parameters (Å^2^)

**Table:**

	*x*	*y*	*z*	*U*_iso_*/*U*_eq_	Occ. (<1)
Br1	0.93272 (9)	0.21923 (16)	0.49100 (14)	0.0729 (5)	0.5214 (19)
C9	0.8583 (5)	0.0318 (10)	0.6902 (8)	0.0531 (17)	0.5214 (19)
H9A	0.8328	0.0122	0.6153	0.064*	0.5214 (19)
H9B	0.8464	−0.0448	0.7381	0.064*	0.5214 (19)
C10	1.1164 (5)	0.0103 (9)	0.7741 (7)	0.0667 (19)	0.5214 (19)
H10	1.1523	−0.0316	0.8307	0.080*	0.5214 (19)
C11	1.0182 (6)	−0.0061 (16)	0.7684 (11)	0.082 (4)	0.5214 (19)
H11	0.9881	−0.0599	0.8208	0.098*	0.5214 (19)
C12	0.9649 (5)	0.0574 (9)	0.6848 (7)	0.057 (2)	0.5214 (19)
C13	1.0089 (4)	0.1363 (8)	0.6063 (6)	0.0555 (17)	0.5214 (19)
C14	1.1077 (4)	0.1516 (9)	0.6101 (7)	0.068 (3)	0.5214 (19)
C15	1.1529 (4)	0.2292 (9)	0.5302 (7)	0.076 (3)	0.5214 (19)
H15	1.1171	0.2707	0.4733	0.092*	0.5214 (19)
C16	1.2513 (5)	0.2448 (9)	0.5352 (7)	0.073 (3)	0.5214 (19)
H16	1.2816	0.2968	0.4816	0.087*	0.5214 (19)
C17	1.3047 (5)	0.1829 (8)	0.6201 (7)	0.068 (3)	0.5214 (19)
H17	1.3707	0.1934	0.6234	0.081*	0.5214 (19)
C18	1.2595 (4)	0.1054 (9)	0.7000 (7)	0.070 (2)	0.5214 (19)
H18	1.2953	0.0638	0.7569	0.085*	0.5214 (19)
C19	1.1611 (4)	0.0897 (9)	0.6950 (6)	0.068 (3)	0.5214 (19)
C9'	0.8450 (6)	0.0553 (11)	0.6468 (9)	0.0531 (17)	0.4786 (19)
H9'1	0.8038	0.0595	0.5800	0.064*	0.4786 (19)
H9'2	0.8458	−0.0359	0.6748	0.064*	0.4786 (19)
C10'	1.0349 (5)	0.2273 (10)	0.4867 (7)	0.0667 (19)	0.4786 (19)
H10'	1.0368	0.2777	0.4209	0.080*	0.4786 (19)
C11'	0.9485 (6)	0.1796 (18)	0.5240 (12)	0.082 (4)	0.4786 (19)
H11'	0.8923	0.1996	0.4842	0.098*	0.4786 (19)
C12'	0.9454 (5)	0.1022 (10)	0.6205 (7)	0.057 (2)	0.4786 (19)
C13'	1.0287 (5)	0.0714 (9)	0.6786 (7)	0.0555 (17)	0.4786 (19)
C14'	1.1158 (4)	0.1209 (10)	0.6427 (7)	0.068 (3)	0.4786 (19)
C15'	1.2003 (5)	0.0901 (10)	0.7016 (8)	0.076 (3)	0.4786 (19)
H15'	1.1986	0.0369	0.7658	0.092*	0.4786 (19)
C16'	1.2875 (6)	0.1393 (10)	0.6641 (9)	0.073 (3)	0.4786 (19)
H16'	1.3438	0.1188	0.7034	0.087*	0.4786 (19)
C17'	1.2901 (6)	0.2191 (10)	0.5678 (8)	0.068 (3)	0.4786 (19)
H17'	1.3482	0.2519	0.5428	0.081*	0.4786 (19)
C18'	1.2056 (5)	0.2498 (9)	0.5089 (7)	0.070 (2)	0.4786 (19)
H18'	1.2073	0.3031	0.4446	0.085*	0.4786 (19)
C19'	1.1184 (4)	0.2007 (9)	0.5463 (7)	0.068 (3)	0.4786 (19)
Br1'	1.02132 (16)	−0.0384 (2)	0.80852 (18)	0.1011 (8)	0.4786 (19)
C1	0.7221 (3)	0.1315 (4)	0.7734 (3)	0.0451 (10)	
C2	0.6685 (3)	0.0219 (5)	0.7571 (4)	0.0515 (11)	
H2	0.6915	−0.0492	0.7149	0.062*	
C3	0.5733 (3)	0.0146 (5)	0.8065 (4)	0.0525 (11)	
C4	0.5388 (3)	0.1316 (5)	0.8710 (4)	0.0531 (11)	
H4A	0.4962	0.1001	0.9288	0.064*	
H4B	0.5020	0.1888	0.8204	0.064*	
C5	0.6191 (3)	0.2159 (4)	0.9280 (3)	0.0471 (10)	
C6	0.6897 (3)	0.2516 (4)	0.8369 (4)	0.0465 (10)	
H6A	0.6598	0.3143	0.7846	0.056*	
H6B	0.7448	0.2954	0.8714	0.056*	
C7	0.6693 (4)	0.1348 (6)	1.0219 (4)	0.0652 (14)	
H7A	0.7186	0.1882	1.0569	0.098*	
H7B	0.6972	0.0558	0.9902	0.098*	
H7C	0.6235	0.1095	1.0772	0.098*	
C8	0.5763 (4)	0.3438 (6)	0.9770 (5)	0.0684 (14)	
H8A	0.6263	0.3970	1.0107	0.103*	
H8B	0.5307	0.3207	1.0332	0.103*	
H8C	0.5450	0.3938	0.9178	0.103*	
O1	0.8111 (2)	0.1500 (3)	0.7355 (3)	0.0642 (10)	
O2	0.5246 (3)	−0.0871 (4)	0.7946 (3)	0.0814 (12)	

### Atomic displacement parameters (Å^2^)

**Table:**

	*U*^11^	*U*^22^	*U*^33^	*U*^12^	*U*^13^	*U*^23^
Br1	0.0660 (7)	0.0816 (11)	0.0705 (9)	0.0125 (6)	−0.0091 (5)	−0.0032 (7)
C9	0.0528 (19)	0.0533 (19)	0.053 (2)	0.0011 (10)	0.0026 (10)	−0.0008 (10)
C10	0.073 (5)	0.065 (5)	0.061 (4)	0.008 (4)	0.007 (4)	0.009 (4)
C11	0.102 (8)	0.075 (9)	0.068 (9)	0.010 (6)	−0.003 (6)	0.013 (6)
C12	0.063 (5)	0.051 (5)	0.060 (6)	0.007 (4)	0.011 (5)	−0.017 (4)
C13	0.040 (4)	0.062 (5)	0.065 (5)	0.001 (3)	0.007 (4)	−0.006 (3)
C14	0.052 (3)	0.067 (6)	0.084 (7)	−0.003 (3)	0.014 (4)	−0.024 (5)
C15	0.055 (7)	0.079 (6)	0.096 (7)	−0.010 (5)	0.012 (5)	−0.004 (5)
C16	0.059 (5)	0.063 (6)	0.096 (7)	0.013 (4)	−0.012 (4)	−0.004 (5)
C17	0.044 (4)	0.067 (6)	0.093 (9)	−0.002 (4)	0.009 (5)	0.008 (6)
C18	0.075 (7)	0.060 (5)	0.077 (6)	−0.001 (5)	0.008 (5)	0.001 (4)
C19	0.050 (5)	0.059 (5)	0.097 (7)	0.004 (4)	0.009 (5)	−0.021 (5)
C9'	0.0528 (19)	0.0533 (19)	0.053 (2)	0.0011 (10)	0.0026 (10)	−0.0008 (10)
C10'	0.073 (5)	0.065 (5)	0.061 (4)	0.008 (4)	0.007 (4)	0.009 (4)
C11'	0.102 (8)	0.075 (9)	0.068 (9)	0.010 (6)	−0.003 (6)	0.013 (6)
C12'	0.063 (5)	0.051 (5)	0.060 (6)	0.007 (4)	0.011 (5)	−0.017 (4)
C13'	0.040 (4)	0.062 (5)	0.065 (5)	0.001 (3)	0.007 (4)	−0.006 (3)
C14'	0.052 (3)	0.067 (6)	0.084 (7)	−0.003 (3)	0.014 (4)	−0.024 (5)
C15'	0.055 (7)	0.079 (6)	0.096 (7)	−0.010 (5)	0.012 (5)	−0.004 (5)
C16'	0.059 (5)	0.063 (6)	0.096 (7)	0.013 (4)	−0.012 (4)	−0.004 (5)
C17'	0.044 (4)	0.067 (6)	0.093 (9)	−0.002 (4)	0.009 (5)	0.008 (6)
C18'	0.075 (7)	0.060 (5)	0.077 (6)	−0.001 (5)	0.008 (5)	0.001 (4)
C19'	0.050 (5)	0.059 (5)	0.097 (7)	0.004 (4)	0.009 (5)	−0.021 (5)
Br1'	0.1561 (17)	0.0705 (11)	0.0769 (14)	−0.0061 (9)	0.0059 (10)	0.0235 (9)
C1	0.043 (2)	0.050 (3)	0.042 (2)	0.0009 (19)	0.0091 (18)	0.0007 (19)
C2	0.051 (3)	0.054 (3)	0.050 (2)	−0.008 (2)	0.008 (2)	−0.009 (2)
C3	0.053 (3)	0.062 (3)	0.042 (2)	−0.017 (2)	−0.001 (2)	0.002 (2)
C4	0.042 (2)	0.064 (3)	0.053 (3)	−0.006 (2)	0.006 (2)	0.001 (2)
C5	0.041 (2)	0.057 (3)	0.043 (2)	−0.003 (2)	0.0087 (18)	−0.001 (2)
C6	0.045 (2)	0.051 (3)	0.044 (2)	−0.0027 (19)	0.0065 (18)	0.0026 (19)
C7	0.072 (3)	0.087 (4)	0.037 (2)	−0.014 (3)	0.001 (2)	0.010 (2)
C8	0.063 (3)	0.073 (3)	0.070 (3)	−0.004 (3)	0.015 (3)	−0.017 (3)
O1	0.0533 (19)	0.059 (2)	0.081 (2)	−0.0126 (15)	0.0272 (17)	−0.0207 (18)
O2	0.069 (2)	0.083 (3)	0.093 (3)	−0.035 (2)	0.018 (2)	−0.018 (2)

### Geometric parameters (Å, º)

**Table:**

Br1—C13	1.907 (6)	C13'—Br1'	1.897 (7)
C9—O1	1.461 (10)	C14'—C15'	1.395 (5)
C9—C12	1.515 (8)	C14'—C19'	1.395 (5)
C9—H9A	0.9700	C15'—C16'	1.395 (5)
C9—H9B	0.9700	C15'—H15'	0.9300
C10—C11	1.383 (6)	C16'—C17'	1.395 (5)
C10—C19	1.387 (6)	C16'—H16'	0.9300
C10—H10	0.9300	C17'—C18'	1.395 (5)
C11—C12	1.382 (6)	C17'—H17'	0.9300
C11—H11	0.9300	C18'—C19'	1.395 (5)
C12—C13	1.376 (6)	C18'—H18'	0.9300
C13—C14	1.390 (6)	C1—C2	1.339 (6)
C14—C19	1.386 (5)	C1—O1	1.345 (5)
C14—C15	1.386 (5)	C1—C6	1.493 (6)
C15—C16	1.386 (5)	C2—C3	1.467 (6)
C15—H15	0.9300	C2—H2	0.9300
C16—C17	1.386 (5)	C3—O2	1.231 (6)
C16—H16	0.9300	C3—C4	1.483 (7)
C17—C18	1.386 (5)	C4—C5	1.548 (6)
C17—H17	0.9300	C4—H4A	0.9700
C18—C19	1.386 (5)	C4—H4B	0.9700
C18—H18	0.9300	C5—C6	1.520 (6)
C9'—O1	1.499 (11)	C5—C8	1.531 (7)
C9'—C12'	1.519 (9)	C5—C7	1.536 (6)
C9'—H9'1	0.9700	C6—H6A	0.9700
C9'—H9'2	0.9700	C6—H6B	0.9700
C10'—C19'	1.379 (7)	C7—H7A	0.9600
C10'—C11'	1.380 (6)	C7—H7B	0.9600
C10'—H10'	0.9300	C7—H7C	0.9600
C11'—C12'	1.383 (6)	C8—H8A	0.9600
C11'—H11'	0.9300	C8—H8B	0.9600
C12'—C13'	1.377 (6)	C8—H8C	0.9600
C13'—C14'	1.389 (7)		
			
O1—C9—C12	109.3 (7)	C14'—C15'—C16'	120.0
O1—C9—H9A	109.8	C14'—C15'—H15'	120.0
C12—C9—H9A	109.8	C16'—C15'—H15'	120.0
O1—C9—H9B	109.8	C15'—C16'—C17'	120.0
C12—C9—H9B	109.8	C15'—C16'—H16'	120.0
H9A—C9—H9B	108.3	C17'—C16'—H16'	120.0
C11—C10—C19	119.7 (4)	C18'—C17'—C16'	120.0
C11—C10—H10	120.2	C18'—C17'—H17'	120.0
C19—C10—H10	120.2	C16'—C17'—H17'	120.0
C12—C11—C10	120.1 (4)	C17'—C18'—C19'	120.0
C12—C11—H11	120.0	C17'—C18'—H18'	120.0
C10—C11—H11	120.0	C19'—C18'—H18'	120.0
C13—C12—C11	120.4 (4)	C10'—C19'—C18'	120.4 (4)
C13—C12—C9	125.5 (6)	C10'—C19'—C14'	119.6 (4)
C11—C12—C9	114.2 (6)	C18'—C19'—C14'	120.0
C12—C13—C14	120.0 (4)	C2—C1—O1	125.7 (4)
C12—C13—Br1	118.9 (4)	C2—C1—C6	123.7 (4)
C14—C13—Br1	121.1 (4)	O1—C1—C6	110.6 (4)
C19—C14—C15	120.0	C1—C2—C3	119.5 (4)
C19—C14—C13	119.6 (3)	C1—C2—H2	120.2
C15—C14—C13	120.4 (3)	C3—C2—H2	120.2
C16—C15—C14	120.0	O2—C3—C2	120.0 (4)
C16—C15—H15	120.0	O2—C3—C4	121.7 (4)
C14—C15—H15	120.0	C2—C3—C4	118.3 (4)
C17—C16—C15	120.0	C3—C4—C5	114.4 (4)
C17—C16—H16	120.0	C3—C4—H4A	108.7
C15—C16—H16	120.0	C5—C4—H4A	108.7
C16—C17—C18	120.0	C3—C4—H4B	108.7
C16—C17—H17	120.0	C5—C4—H4B	108.7
C18—C17—H17	120.0	H4A—C4—H4B	107.6
C17—C18—C19	120.0	C6—C5—C8	109.8 (4)
C17—C18—H18	120.0	C6—C5—C7	110.2 (4)
C19—C18—H18	120.0	C8—C5—C7	110.0 (4)
C14—C19—C18	120.0	C6—C5—C4	107.1 (3)
C14—C19—C10	120.2 (3)	C8—C5—C4	109.5 (4)
C18—C19—C10	119.8 (3)	C7—C5—C4	110.2 (4)
O1—C9'—C12'	104.8 (7)	C1—C6—C5	112.2 (3)
O1—C9'—H9'1	110.8	C1—C6—H6A	109.2
C12'—C9'—H9'1	110.8	C5—C6—H6A	109.2
O1—C9'—H9'2	110.8	C1—C6—H6B	109.2
C12'—C9'—H9'2	110.8	C5—C6—H6B	109.2
H9'1—C9'—H9'2	108.9	H6A—C6—H6B	107.9
C19'—C10'—C11'	120.5 (4)	C5—C7—H7A	109.5
C19'—C10'—H10'	119.8	C5—C7—H7B	109.5
C11'—C10'—H10'	119.8	H7A—C7—H7B	109.5
C10'—C11'—C12'	120.1 (4)	C5—C7—H7C	109.5
C10'—C11'—H11'	120.0	H7A—C7—H7C	109.5
C12'—C11'—H11'	120.0	H7B—C7—H7C	109.5
C13'—C12'—C11'	119.9 (4)	C5—C8—H8A	109.5
C13'—C12'—C9'	127.3 (7)	C5—C8—H8B	109.5
C11'—C12'—C9'	112.8 (7)	H8A—C8—H8B	109.5
C12'—C13'—C14'	120.4 (4)	C5—C8—H8C	109.5
C12'—C13'—Br1'	118.4 (4)	H8A—C8—H8C	109.5
C14'—C13'—Br1'	121.2 (4)	H8B—C8—H8C	109.5
C13'—C14'—C15'	120.5 (4)	C1—O1—C9	116.1 (4)
C13'—C14'—C19'	119.5 (4)	C1—O1—C9'	117.1 (4)
C15'—C14'—C19'	120.0	C9—O1—C9'	23.1 (5)
			
C19—C10—C11—C12	−1 (2)	C12'—C13'—C14'—C19'	−0.8 (14)
C10—C11—C12—C13	1 (2)	Br1'—C13'—C14'—C19'	179.6 (6)
C10—C11—C12—C9	−179.1 (13)	C13'—C14'—C15'—C16'	179.4 (11)
O1—C9—C12—C13	−75.3 (11)	C19'—C14'—C15'—C16'	0.0
O1—C9—C12—C11	104.4 (12)	C14'—C15'—C16'—C17'	0.0
C11—C12—C13—C14	0.6 (17)	C15'—C16'—C17'—C18'	0.0
C9—C12—C13—C14	−179.8 (9)	C16'—C17'—C18'—C19'	0.0
C11—C12—C13—Br1	178.9 (11)	C11'—C10'—C19'—C18'	−179.5 (12)
C9—C12—C13—Br1	−1.5 (13)	C11'—C10'—C19'—C14'	2.4 (17)
C12—C13—C14—C19	−1.6 (12)	C17'—C18'—C19'—C10'	−178.1 (10)
Br1—C13—C14—C19	−179.8 (5)	C17'—C18'—C19'—C14'	0.0
C12—C13—C14—C15	178.9 (7)	C13'—C14'—C19'—C10'	−1.3 (11)
Br1—C13—C14—C15	0.7 (11)	C15'—C14'—C19'—C10'	178.1 (10)
C19—C14—C15—C16	0.0	C13'—C14'—C19'—C18'	−179.4 (11)
C13—C14—C15—C16	179.5 (10)	C15'—C14'—C19'—C18'	0.0
C14—C15—C16—C17	0.0	O1—C1—C2—C3	178.3 (4)
C15—C16—C17—C18	0.0	C6—C1—C2—C3	−2.0 (7)
C16—C17—C18—C19	0.0	C1—C2—C3—O2	−178.0 (5)
C15—C14—C19—C18	0.0	C1—C2—C3—C4	1.4 (7)
C13—C14—C19—C18	−179.5 (10)	O2—C3—C4—C5	151.0 (4)
C15—C14—C19—C10	−179.0 (10)	C2—C3—C4—C5	−28.4 (6)
C13—C14—C19—C10	1.5 (10)	C3—C4—C5—C6	52.9 (5)
C17—C18—C19—C14	0.0	C3—C4—C5—C8	171.8 (4)
C17—C18—C19—C10	179.0 (9)	C3—C4—C5—C7	−67.0 (5)
C11—C10—C19—C14	−0.4 (15)	C2—C1—C6—C5	29.6 (6)
C11—C10—C19—C18	−179.4 (11)	O1—C1—C6—C5	−150.7 (4)
C19'—C10'—C11'—C12'	−1 (2)	C8—C5—C6—C1	−170.9 (4)
C10'—C11'—C12'—C13'	−1 (2)	C7—C5—C6—C1	67.8 (5)
C10'—C11'—C12'—C9'	−178.3 (14)	C4—C5—C6—C1	−52.1 (5)
O1—C9'—C12'—C13'	83.4 (12)	C2—C1—O1—C9	−11.2 (7)
O1—C9'—C12'—C11'	−99.3 (13)	C6—C1—O1—C9	169.1 (5)
C11'—C12'—C13'—C14'	1.8 (18)	C2—C1—O1—C9'	14.7 (8)
C9'—C12'—C13'—C14'	178.9 (10)	C6—C1—O1—C9'	−165.1 (6)
C11'—C12'—C13'—Br1'	−178.6 (12)	C12—C9—O1—C1	−162.9 (5)
C9'—C12'—C13'—Br1'	−1.5 (15)	C12—C9—O1—C9'	98.9 (13)
C12'—C13'—C14'—C15'	179.8 (8)	C12'—C9'—O1—C1	179.2 (6)
Br1'—C13'—C14'—C15'	0.2 (12)	C12'—C9'—O1—C9	−87.4 (13)
